# Generation of Photopolymerized Microparticles Based on PEGDA Hydrogel Using T-Junction Microfluidic Devices: Effect of the Flow Rates

**DOI:** 10.3390/mi14071279

**Published:** 2023-06-21

**Authors:** Gabriela Hinojosa-Ventura, Mario Alberto García-Ramírez, José Manuel Acosta-Cuevas, Orfil González-Reynoso

**Affiliations:** 1 Chemical Engineering Department, CUCEI, Universidad de Guadalajara, Blvd.M. García Barragán # 1451, Guadalajara 44430, Jalisco, Mexico; gabriela.hinojosa@academicos.udg.mx (G.H.-V.); jose.acosta@alumnos.udg.mx (J.M.A.-C.); 2 Electronics Department, CUCEI, Universidad de Guadalajara, Blvd.M. García Barragán # 1451, Guadalajara 44430, Jalisco, Mexico

**Keywords:** microfluidic devices, T-junction geometry, dispersed phase flow (Qd), continuous phase flow (Qc), entrapped drugs, PEGDA hydrogel microparticles (*MPs*)

## Abstract

The formation of microparticles (*MPs*) of biocompatible and biodegradable hydrogels such as polyethylene glycol diacrylate (PEGDA) utilizing microfluidic devices is an attractive option for entrapment and encapsulation of active principles and microorganisms. Our research group has presented in previous studies a formulation to produce these hydrogels with adequate physical and mechanical characteristics for their use in the formation of *MPs*. In this work, hydrogel *MPs* are formed based on PEGDA using a microfluidic device with a T-junction design, and the *MPs* become hydrogel through a system of photopolymerization. The diameters of the *MPs* are evaluated as a function of the hydrodynamic condition flow rates of the continuous (Qc) and disperse (Qd) phases, measured by optical microscopy, and characterized through scanning electron microscopy. As a result, the following behavior is found: the diameter is inversely proportional to the increase in flow in the continuous phase (Qc), and it has a significant statistical effect that is greater than that in the flow of the disperse phase (Qd). While the diameter of the *MPs* is proportional to Qd, it does not have a significant statistical effect on the intervals of flow studied. Additionally, the *MPs’* polydispersity index (PDI) was measured for each experimental hydrodynamic condition, and all values were smaller than 0.05, indicating high homogeneity in the *MPs*. The microparticles have the potential to entrap pharmaceuticals and microorganisms, with possible pharmacological and bioremediation applications.

## 1. Introduction

### 1.1. Application of Hydrogel MPs

The hydrogel *MPs* have been used in several biotechnological applications, such as cellular entrapment and encapsulation, tissue engineering, and systems of pharmaceutical administration. In this last case, it has been observed that with the use of hydrogels in the form of *MPs*, these have a high surface-volume ratio and can be delivered within microscale structures such as microblood vessels and tissues [1]. Hydrogels are materials that fulfill the required conditions for their use as vehicles in the administration of drugs, such as biodegradability, biocompatibility, low antigenicity, and the capability of responding to stimuli (to pH, temperature, and the variations of ionic force) and maintaining their composition [2,3]. 

Structurally, they are nets of crosslinked polymers that have the capacity of contracting and expanding through the retention or liberation of water [1,2]. Hydrogels are also utilized because of their good physical, mechanical, and biological properties; alternatively, the pore size and hydrophobicity of the hydrogels can be adjusted and easily improved in the laboratory [1,2,4,5]. The reticulation of hydrogels can be performed through different mechanisms, such as thermal, ionic, and photopolymerization (the mechanism will depend on the material and the application) [1,6].

### 1.2. Application and Uses of PEG

One of the materials for the formation of hydrogels with the greatest number of pharmaceutical, biomedical, and biotechnological applications is the polyethylene glycol monomer (PEG). The pioneer in the use of PEG was Abuchowski et al. (1977), who demonstrated the usefulness of the conjunction of a protein with PEG. They found improved immunogenicity, better solubility, and a longer plasma half-life [7]. Finding the mentioned properties, many active principles are conjugated with PEG and are available in the market, such as the enzymes adenosine deaminase (ADA), L-asparaginase, interferon α2b (IFN-2b), interferon α2a (IFN-2a), granulocyte colony-stimulating factor (G-CSF), HGH receptor antagonist, anti-VEGF aptamer, epoetin beta, anti-TNF Fab′, peginterferon beta-1a, naloxone, factor VIII, and phenylalanine ammonia lyase [8,9,10,11,12,13,14,15,16,17,18,19,20,21,22]. Therefore, PEG is a material with a high potential for use as a vehicle for the entrapment of active principles and their posteriorly controlled liberation.

### 1.3. Importance of MPs´ Formation Using Microfluidic Devices

The formation of *MPs* through reticulation in batches produces sizes with a high polydispersity, which makes their application difficult. Xu et al. (2009) made a comparison between *MPs* produced by emulsion in batch systems employing the polymeric matrix of lactic-co-glycolic acid (PLGA) charged with bupivacaine and the microparticles produced through microfluidic devices. The results showed that the release rate of *MPs* produced through the traditional method (polydisperse *MPs*) is less than that of *MPs* produced through microfluidic devices (monodisperse *MPs*), taking as a reference the same average size for both processes. In addition, the kinetics of the release of monodisperse *MPs* are more constant than those of polydisperse *MPs* (release bursts of the pharmaceutical are observed in the first hour) [23]. Therefore, it is considered that a more uniform distribution of particle size presents better control over the release of entrapped or encapsulated pharmaceuticals and high efficiency in the encapsulation [24].

For the reasons outlined above, the formation of *MPs* through microfluidic devices is a better alternative than the traditional methods of batching. It has been demonstrated that through this technology, homogeneous sizes are obtained in the synthesized *MPs* or nanoparticles (NPs), and when the index value of polydispersity of *MPs* is under 0.05, they are considered homogeneous [25,26,27,28]. Other advantages in the use of microfluidic devices are better control over the size, shape, and frequency, as well as continuous production, reproducibility of conditions, and extension of the process through parallelization of the production [29,30,31,32,33,34,35].

The most common geometries of microdevices for the formation of microdrops are the T-junction, co-flow, flow-focusing, and droplet formation using capillary unions [1,36]. The technology of microfluids consists of introducing flows of immiscible fluids within the channels of the device (on a micrometric scale). When introducing both fluids to the device, the regular rupture of one of the phases (the disperse phase) takes place in the other phase (the continuous phase), and the resulting emulsion, also called a droplet, occurs under the equilibrium between the inertia forces, the viscous forces, and the interfacial tension between both fluids [37,38,39].

The drop generation, size, shape, and number of *MPs* depend on the properties and flow rates of the fluids in the continuous phase and the dispersed phase (viscosity, density, and surface tension), as well as the device characteristics and its channels (design, material geometry, surface properties, and roughness) [33,34,35]. Since there are multiple factors involved in the formation of droplets and/or *MPs*, specifically in relation to the generation of PEGDA hydrogel particles, researchers have carried out studies that involve several of the mentioned aspects (Table 1).

In Table 1, it can be observed that none of these studies have used eosine Y as a photo-initiator to start the curing process [38,40,41,42,43,44]. In this work, eosin Y is used as the photo-initiator, which needs light with a wavelength of about 520 nm, or visible light [2,45,46]. Under these conditions, neither the materials nor the microorganisms are at risk of being harmed by UV light irradiation. 

In the formation of *MPs* by microfluidics, each investigation presents its own conditions due to the many different materials utilized for the dispersed phase fluid in the synthesis of *MPs*, as well as the design and material of the device used; thus, a specific flow range should be established for the system used. In one previous study, our research group established a formulation for a hydrogel based on PEGDA with good physical and mechanical properties [46]. In the present work, this pre-polymeric solution was used to form *MPs* in a resin device (T-junction design) and adapted to a green light photopolymerization area. In the device-fluids system, a zone for the generation of spherical microparticles of various sizes is established, and the simultaneous effects of dispersed and continuous phase flows on the size and number of the microparticles produced are evaluated by a 2^2^ factorial design. 

## 2. Materials and Methods

### 2.1. Materials 

A transparent 3D impression resin bought from Anicubic Technology Co. Ltd. was used to make the microfluidic device. For the formation of *MPs* in hydrogels, the following compounds were used: 1-vinil-2-pyrrolidone (NVP); eosin Y; triethanolamine (TEA); and polyethylene glycol diacrylate (PEGDA). Those mentioned compounds were purchased from Sigma-Aldrich, St. Louis, MO, USA. Mineral oil (Golden Bell) was used as a carrier of droplets and *MPs*, which in this paper is called the continuous phase. The solution of hydrogel was prepared according to the formulation H5 made by Acosta-Cuevas et al. (2021), which contains, as monomer and crosslinker, the PEGDA of 575 Da (Mw) (670 Mm); as monomer and accelerator, the NVP at 37 mM; as photoinitiator, eosin Y (0.005 mM); and, finally, as co-initiator, the TEA at 225 mM. All the solutions were kept in amber-colored tubes to avoid contact with light and thus premature reticulation [46]. 

### 2.2. Design and Impression of Microdevices

**Figure 1:**
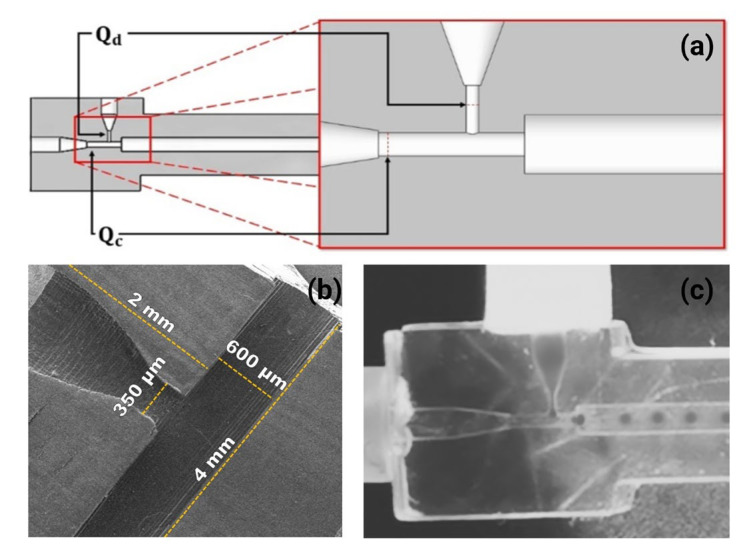
(**a**) T-junction microfluidic device designed in SolidWorks software with two inputs (Qd and Qc). (**b**) SEM image of the chip’s structures (length: 4mm, width: 600 µm, depth: 350 µm). (**c**) Experiment with setup, breaking Qd with Qc.

The microfluidic device used in this research was designed in SolidWorks software with two inlet channels corresponding to Qc and Qd, respectively, where Qc is the flow in the main channel and crosses with Qd, which flows in a secondary channel (Figure 1a). Figure 1b shows the chip’s structures, which are 4 mm in length, 600 µm in width, and 350 µm in depth. The material used for making it was a transparent resin, Anycubic Tough Clear (Anycubic, Kowloon, China), using an Elegoo Mars Pro 3D printer (Elegoo, Shenzhen, China). The microdevice was printed in 20 min at a wavelength of 405 nm. Then the channels of the device were washed three times with isopropyl alcohol and dried for its posterior use. In Figure 1c, the generation of the drop or emulsion is observed by breaking Qd with Qc. 

### 2.3. Synthesis of the MPs in the Microfluidic Device

**Figure 2:**
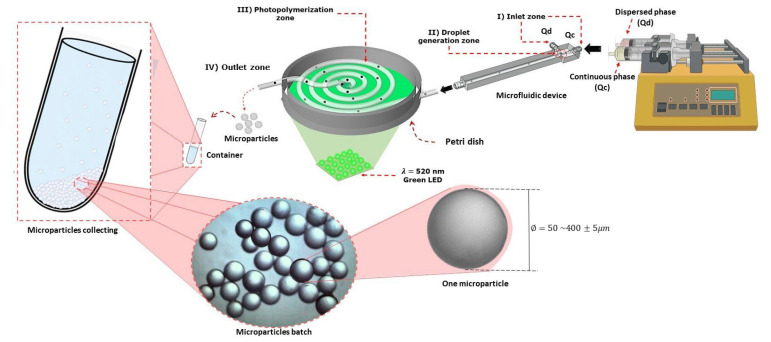
Representation of the procedure that was carried out for the synthesis of the spherical *MPs* of PEGDA hydrogel in a T-junction device and polymerized by green light.

Figure 2 shows a methodology schematic of all methodologies used for the synthesis of the spherical *MPs* of PEGDA hydrogel. The T-junction device (Figure 1) is shown with two input channels corresponding to the flows: the continuous phase (Qc input channel) and the dispersed phase (Qd input channel). Those fluids were placed in syringes (Hamilton) and were adapted to an Inovenso Pump System IPS-14 under different conditions of flow (stage I of Figure 2).

The fluxes of Qd and Qc enter the device with each input, and they intersect to form the drop; the continuous phase acts as the carrier of the drop corresponding to Qd. For the formation of the drop, both fluids mentioned must have different properties and be immiscible with each other (stage II of Figure 2).

To photopolymerize the generated droplets of the disperse phase in the microfluidic device, a polymerization zone was adapted, which consisted of a masterflex^®^ hose with an internal diameter of 8 × 10^−4^ m and 0.3 m of length in the form of a serpentine. In this photopolymerization zone, a lamp of 50 W was adapted with an approximate wavelength of 520 nm, corresponding to visible light. In this zone, the reaction of reticulation of the drop of the previously formed PEGDA hydrogel polymer in the device in Figure 1 was carried out. The reaction triggers due to the absorption of the light photons by the photoinitiator eosin Y, which transforms into the excited state, and then it reacts with TEA to generate primary radicals. The primary radicals propagate by reaction with NVP and PEGDA. The propagation of radicals through PEGDA results in the formation of double pendant bonds in the polymeric chains. The reaction of crosslinking implies propagation through double pendant bonds that lead to the formation of gel [45,46,47] (stage III of Figure 2).

Each sample was collected in Falcon tubes of 15 mL with a volume of water of 5 mL (stage IV). Following, the oily phase was removed with a Pasteur pipette from the upper part of the tube, and those were washed with a solution of Tween 80 at 0.5% repeatedly until removing the remnants of oil in the *MPs*. In each of the washings, the supernatant was removed after centrifugating for 5 min at 5000 rpm [41]. Next, the samples were dried for the measurement of the diameters and their characterization.

### 2.4. *Evaluation of the Diameter, Number, and Polydispersity Index of the MPs*

Sizes of diameters, amounts (average), and PDI of microparticles were assessed under flows of 5 and 10 µL/min as well as 300 and 400 µL/min of Qd and Qc, respectively, in the microfluidic device described previously. The *MPs*´ samples were taken from an aqueous solution and placed on a slide to dry. Afterwards, samples were observed in the Motic optical microscope, and the images were obtained to carry out the determination of the diameters in the Motic Images Plus software 2.0 ML, which was previously calibrated at the required length intervals. The averages of the diameters of more than 100 spherical *MPs* corresponding to each treatment were obtained.

After this, the results obtained were analyzed in SPSS and Statgraphics software. To evaluate the number of *MPs* produced under the different flow conditions mentioned above, Equation (1) calculated the average volume of spherical *MPs* per treatment $\;({\bar{V}}_{MPs}$) with the sphere formula $\;({\bar{V}}_{MPs}=\frac{4\pi {\bar{r}}^{3}}{3})$, where ${\bar{r}}^{3}\;$ is the average radius (units) per treatment of spherical *MPs* that was obtained from the previously calculated average diameters (considering the reduction in the diameter for the evaporation of the water).

For each treatment, the total volume spent on the dispersed phase ($\;V_{DP}$) was 100 µL. Since the *MPs* are formed by the breaking of the flow of the dispersed phase with the continuous phase, the number of *MPs* produced per treatment ($\bar{N}o_{MPs}$) is represented in Equation (1), which is the quotient between the volume spent in the dispersed phase ($\;V_{DP}$) and the average volume of spherical *MPs* per treatment (obtained from the sphere formula).

(1) $$\bar{N}o_{MPs}=\frac{V_{DP}}{{\bar{V}}_{MPs}}$$

Next, the samples were taken to be characterized through a scanning electron microscope (SEM). The polydispersity index (PDI), also known as the polydispersity or heterogeneity index, is a measure of the degree of uniformity of a distribution of particle sizes. Therefore, monodispersity defines the degree of uniformity of the *MPs*, and PDI is represented in Equation (2), where *St Desv* is the standard deviation of the diameter and the size of the mean is the mean of the diameters. The value of PDI is utilized as the criteria to determine if the system presents a monodisperse pattern; if the value is under 0.05, the particles are considered homogeneous and/or have little size variation with respect to each other [25,48]. Another way to validate the monodisperse patterns of *MPs* is the coefficient of variation (CV), that is, *St Desv* divided by the size of the media: $CV=\frac{St\;Desv}{Size\;media}$. The criteria for having a monodispersity size distribution is CV < 5% [26,40,42,44].

(2) $$PDI={\left(\frac{St\;Desv}{Size\;media\;}\right)}^{2}$$

### 2.5. Experimental Design and Statistical Analysis

The hydrodynamic conditions of Qd and Qc were analyzed by a T-junction microdevice. For the statistical analysis, an experimental design 2^2^ was carried out with a repetition (n = 2); the tested fluids were Qd of 5 and 10 µL/min of prepolymeric solution of PEGDA hydrogel (formulation H5) and Qc of 300 and 400 µL/min of mineral oil. An ANOVA was carried out in the Stratigraphic Centurion^®^ 19 software. In addition, a comparison of independent treatments was made using Tukey’s test in the SPSS program. To analyze the size distribution of *MPs* for the different treatments, we used the statistical program SPSS with several repetitions (n) greater than 100.

## 3. Results

### 3.1. Synthesis of MPs of PEGDA Hydrogel in the Microfluidic Device 

The formulation called H5 showed the best physical and mechanical properties in terms of resistance in preliminary research described by Acosta-Cuevas et al. (2021). This prepolymeric solution was used in the present work as a dispersed phase for the formation of *MPs*. Mineral oil, hexadecane, and silicone oil have previously been utilized as a continuous phase to transport PEGDA hydrogel *MPs*, with mineral oil being the most commonly used; hence, in this work, mineral oil was used as the continuous phase [38,40,41,42,44,47]. 

To assess the impact of the Qd and Qc flows on the diameter of *MPs*, the fluids and their compositions in this study were kept constant. The diameters of the *MPs* from the various treatments were measured in the program Motic Images Plus 2.0. Later, the Statgraphics software was used to examine the findings of the statistical design. 

The devices have specific features such as channel diameters, roughness, design, channel shape, etc. Likewise, it is typical for each investigation to establish the concentrations and formulations that make up the dispersed phase; consequently, it is necessary to establish the operational conditions for each system. In the current work, the production of spherical *MPs* is looked at; therefore, a drip regime is required. Thus, the minimum limit of Qd was identified when the pressure of Qc blocked the generation of the drop, and the maximum limit was observed when Qd and Qc allowed the formation of a spherical droplet with a similar diameter to the outlet channel (when the flow of Qd was increased above the maximum limit, ovoid shapes were generated). 

In terms of Qc, the minimum limit happens when the flow allows Qd droplets to invade its channel and those deform from spherical to ovoid shape (when the Qc was lower than the minimum limit, it obstructed the channels). On the other hand, Qc is inversely proportional to the diameter of the droplets, so the maximum flow of Qc is dictated by the decrement or null generation of droplets. For statistical analysis, the Qd and Qc flows were established within these operating limits, where spherical droplets were obtained.

**Figure 3:**
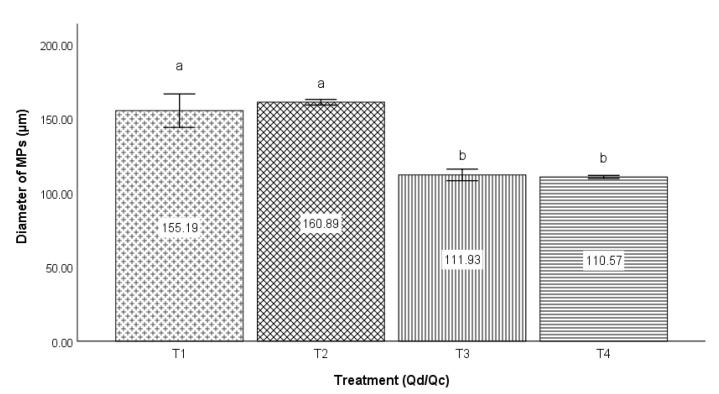
Effect of Qd and Qc on the size of the diameters of the *MPs* produced in a microfluidic device and photopolymerized by green light; the values of each bar represent the means ± SD of two measurements. The letters located over the error bars show significance: different letters indicate the existence of significant differences between the compared groups, and identical letters indicate the absence of significance (Tukey, *p* ≤ 0.05).

In Figure 3, the results of average diameters from the experimental 2^2^design are shown, where 5 and 10 µL/min are the low and high levels of Qd, respectively, and 300 and 400 µL/min are the low and high levels of Qc, respectively. There are four treatments, with their corresponding standard deviations. Thus, treatment one (T1) corresponds to 5 and 300 μL/min of the fluxes of Qd and Qc, treatment two (T2) to 10 and 300 μL/min of Qd and Qc, treatment three (T3) to 5 and 400 μL/min of Qd and Qc, and treatment four (T4) to 10 and 400 μL/min of Qd and Qc, respectively.

The bar graph corresponding to Figure 3 shows the comparison between the four treatments with respect to the average diameter of the *MPs*; the comparison between T1 and T2 (155.19 and 160.00 µm), as well as between T3 and T4 (111.93 and 110.57 µm), did not observe a significant difference in the diameter of the *MPs*; that is, the flow of Qd did not significantly impact the diameter of those particles. Contrary to the above, in the cases of T1 and T3 (155.19 and 111.93 µm), T1 and T4 (155.19 and 110.57 µm), T2 and T3 (160.89 and 111.93 µm), as well as T2 and T4 (160.89 and 110.57 µm), a significant difference was observed in the diameters of the *MPs*; that is, the effect of the Qc flow rate was a significant factor in the diameter of the *MPs* obtained.

Experimental design 2^2^ was used to evaluate the effect of two factors and their interaction effect; this is represented as design 2^k^, where the number 2 represents the levels (a low and a high) and the letter k represents the factors, because the exponent two indicates that there are two factors. In the present work, Qd and Qc are the factors; for Qd, the levels low and high are 5 and 10 µL/min, respectively, and for Qc, the levels low and high are 300 and 400 µL/min, respectively.

**Figure 4:**
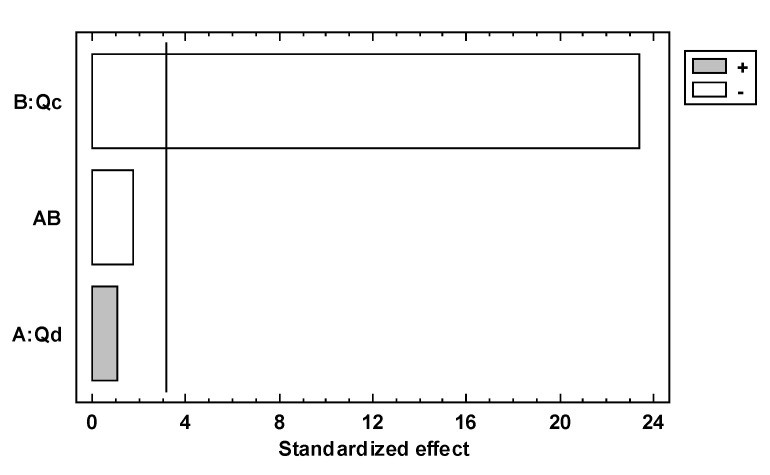
Standardized diagram for the diameter as a response variable and Qd and Qc dependent variables. The significance level is denoted by an alpha of 0.05 (α = 0.05).

Pareto’s standardized diagram, Figure 4 and Figure 6, is a graphic depiction resulting from experimental design 2^2^, which is used to find the factor and the interaction effects that are most important in the response variables. This diagram has a reference line where any effect that extends past this reference line is potentially important or has a significant effect, and the length of the bar is proportional to the effect on the response variable. Additionally, this diagram has a plus sign that means the effect of the factor is proportional to the response variable or has a positive effect on the response variable, and in contrast to that, the minus sign means the effect of the factor is inversely proportional to the response variable or has a negative effect on the response variable.

The standardized effect is on the abscissa axis in Figure 4, and the factors Qd and Qc are on the ordinate axis. The bars represent the standardized effects of Qd and Qc, and thus the white bar corresponding to the Qc factor exceeds the reference line, indicating that the Qc factor is statistically significant and has a negative effect on *MPs´* diameters.

The tendency of the MPs´ diameters regarding Qc is that the diameter of the *MPs* is inversely proportional to the increase in the flow of Qc; this is represented in Equation (3), and these results are consistent with those of other authors [33,34,35,42,43,49]. However, with this study, we can observe that Qc is the most important factor in relation to the diameter of *MPs*.

(3) $$D_{MPs}\propto \;\left(\frac{1}{Q_{c}}\right)$$

Figure 4 also shows that the gray bar corresponding to Qd does not exceed the line of the reference, which indicates that Qd is not statistically significant. The gray bar shows a positive effect on the diameter of *MPs*, which means that the flow of Qd is proportional to the diameter. The tendency of the *MPs*´ diameters regarding Qd is shown in Equation (4), and this tendency has been reported by other authors [38,42,49]. The interaction bar of both factors does not show a statistically significant effect on this response variable.

(4) $$D_{MPs}\propto \;Q_{d}$$

**Figure 5:**
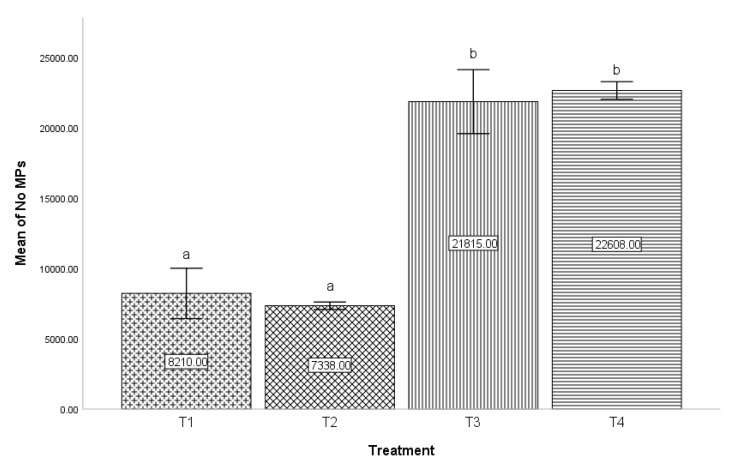
Effect of Qd and Qc on the mean number of *MPs* produced in a microfluidic device and photopolymerized by green light. Values represent the means ± SD of two measurements. The letters located over the error bars show significance: different letters indicate the existence of significant differences between the compared groups, and identical letters indicate the absence of significance (Tukey, *p* ≤ 0.05).

In the bar graph corresponding to Figure 5, the comparison between the four treatments is represented with respect to the number of *MPs* produced in each volume for the different treatments described above (0.1 mL of dispersed phase). The water loss of the hydrogel microparticles is considered for the calculations, which was 60%; these data were reported in the work that preceded this investigation by Acosta-Cuevas et al. (2021). In the comparison between T1 and T2 (8210 and 7338 *MPs*, respectively), as well as in T3 and T4 (21,815 and 22,608 *MPs*, respectively), there was no significant difference in the number of *MPs* produced; that is, the Qd flow did not significantly impact the amount produced. Contrary to the above, between T1 and T3 (8,210 and 21,815 *MPs*), T1 and T4 (8210 and 22,608 *MPs*), T2 and T3 (7338 and 21,815 *MPs*), as well as T2 and T4 (7338 and 22,608 *MPs*), a significant difference was observed in the diameters of the *MPs*; that is, the effect of the flow of Qc was significant in the number of *MPs* produced, considering a constant volume of the dispersed phase for all treatments.

**Figure 6:**
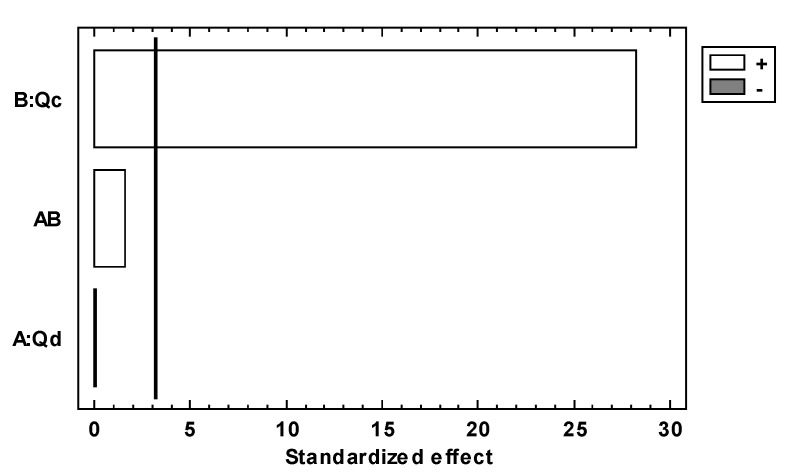
Pareto’s standardized diagram for the number of *MPs* produced as a response variable and Qd and Qc dependent variables (α = 0.05).

Figure 6 shows Pareto’s standardized diagram for the number of *MPs* produced as a dependent variable. Figure 6 shows that both factors Qd and Qc have a positive standardized effect (white colors), which means that the fluxes of Qd and Qc have a positive effect on the number of *MPs* and both factors are proportional to the number of *MPs* produced. With respect to the Qc factor, it is observed that the bar of Qc exceeds the reference line, which means that this factor is statistically significant in the number of *MPs* produced, but the bar of Qd does not exceed the reference line, which means that Qd is not statistically significant in the number of *MPs* produced. The interaction bar of both factors does not show a statistically significant effect on this response variable.

### 3.2. Measuring and Characterization of the Size of the MPs through Optical Microscopy and Scanning Electron Microscopy, and Evaluation of the Polydispersity Index

As has been previously argued in this work, the utilization of microfluidic devices has as its main advantage the production of *MPs* with high homogeneity and low values of PDI. The *MPs* from the different treatments were measured in the software Motic Images Plus 2.0 ML, from which the diameter data was obtained. Subsequently, the data was input into the statistical program SPSS to obtain the size distribution of *MPs* from each of the treatments; an n greater than 100 was used.

**Figure 7:**
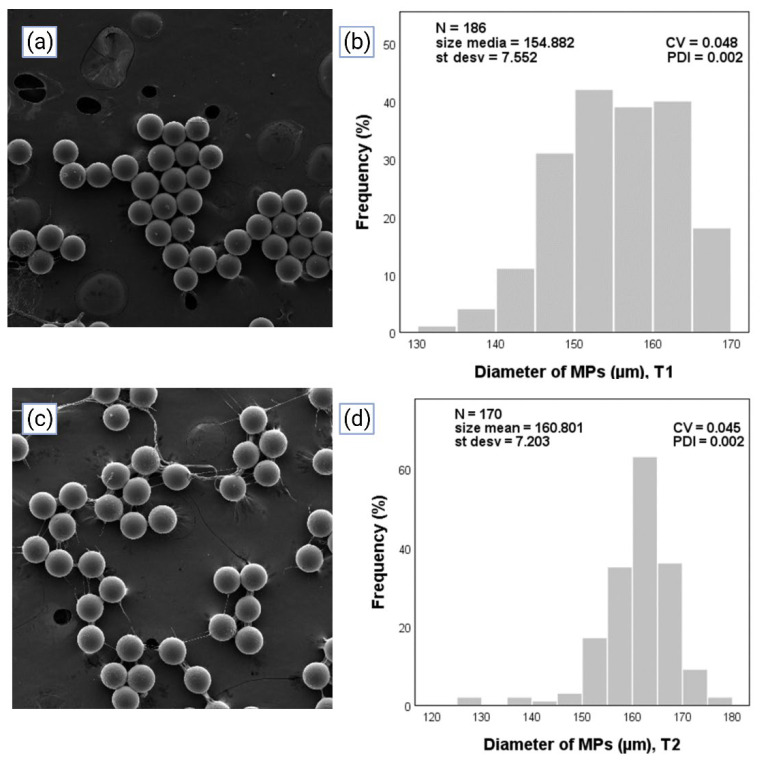
*MPs* generated in the microfluidic device were adapted to green light photopolymerization in a T-junction device. (**a**) SEM images of *MPs* corresponding to T1. (**b**) Image of the representation of the size distribution of *MPs* in T1. (**c**) SEM images of *MPs* corresponding to T2. (**d**) Image of the representation of the size distribution of *MPs* in T2.

**Figure 8:**
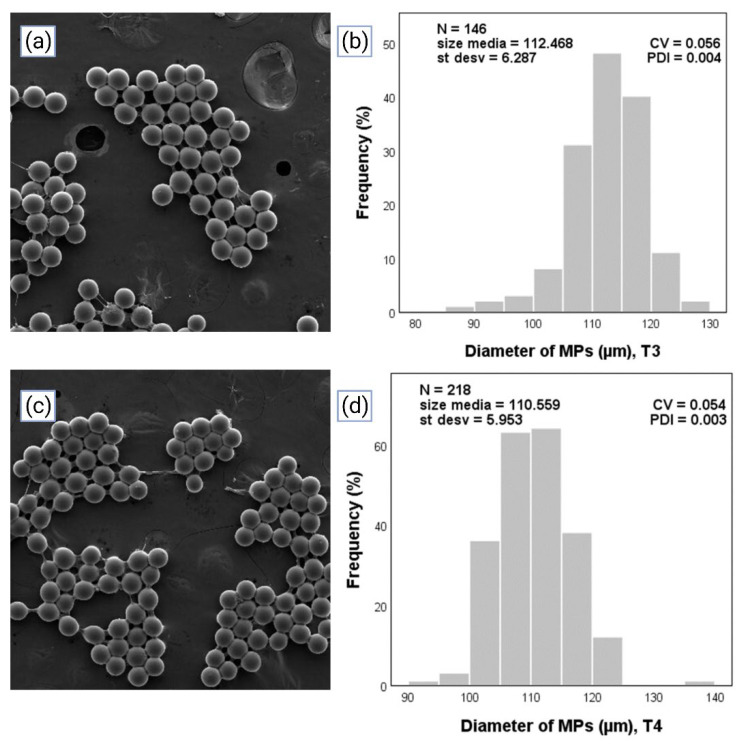
*MPs* generated in the microfluidic device were adapted to a green light photopolymerization system in a dripping regime. (**a**) SEM images of *MPs* corresponding to T3. (**b**) Image of the representation of the size distribution of *MPs* in T3. (**c**) SEM images of *MPs* corresponding to T4. (**d**) Image of the representation of the size distribution of *MPs* in T4.

The results of the different treatments are observed in Figure 7 and Figure 8. On the left are images corresponding to SEM images, and on the right are graphics representing the size distribution of *MPs,* where N is the number of *MPs* counted, size media is the mean of diameters, *St desv* (%) is the standard deviation of the diameter, and PDI is the value of the polydispersity index; in the abscissa is the diameter (µm) and in the ordinate is the frequency (%). With the obtained statistical data, the PDI was calculated with Equation (3) based on the criteria of considering *MPs* homogeneous when the value of the PDI was lower than 0.05 [48].

PDI values of 0.002, 0.002, 0.004, and 0.003 were observed for the relationships of 5/300, 10/300, 10/400, and 5/400 215 of Qd/Qc, respectively. In Figure 7 and Figure 8, regardless of the treatment, it was shown that all the values of PDI were lower than 0.05, therefore meeting the criteria mentioned above. Therefore, PEGDA monodisperse hydrogel *MPs* for the different treatments in a microfluidic T-junction device under a controlled drop regime were produced. The values of T1 and T2 are below the criteria, but T3 and T4 meet it.

## 4. Conclusions

Technology through microfluidic devices is a good option to produce *MPs* due to its easy operation and stable results. However, for each system, it is required to establish specific flows for *MPs´* formation. In this work, the effects on the diameter and frequency of *MPs* were studied by changing the Qd and Qc simultaneously. Results established experimental strategies in such a way to obtain *MPs* with desired characteristics such as size, spherical shape, or production yields.

With respect to the experimental design, where the effects of Qc and Qd were evaluated for both the diameter and number of *MPs* produced, Qc showed a statistically significant effect, and Qd had no statistically significant effect.

Since the trends of each of the flows are known, it is possible to determine the exact flows of both to achieve the desired diameter through linear regression of the experimental data examined with statistical analysis 2^2^.

A method of photopolymerization with visible light was successfully used for drop gelation produced through microdevices that later became *MPs* of PEGDA hydrogel. Using this photopolymerization technique, drugs and microorganisms can be entrapped without incurring the risk of suffering UV light damage.

Finally, *MPs* were obtained through microfluidic devices with polydispersity indexes of under 0.05; therefore, it is considered that monodisperse *MPs* were obtained. These results are considered promising for future research since the variation in sizes hinders research in biological systems. With homogeneous *MPs´* sizes, it can be possible to proceed with research related to the release of controlled pharmaceuticals and later with tests in trials in vitro or in vivo.

## Figures and Tables

**Table 1:** Photopolymerized microparticles based on PEGDA hydrogel using microfluidic devices.

Continuous Phase	Polymerization Light	Channel Width	Device Design	Material	Reference
Mineral oil	UV-light	40	T-junction	PDMS	[40]
Mineral oil	UV-light	100	Flow-focusing (MFFD)	PDMS	[38]
Mineral oil	UV-light	50	Flow-focusing (MFFD)	PDMS	[41]
Hexadecane	UV-light	200	Flow-focusing (MFFD)	PDMS	[42]
Silicone oil	UV-light	75	T-junction	PDMS	[43]
Silicone oil	UV-light	107–400	Flow-focusing (MFFD)	Glass capillary	[44]

## References

[1] Moreira A., Carneiro J., Campos J.B.L.M., Miranda J.M. (2021). Production of Hydrogel Microparticles in Microfluidic Devices: A Review. Microfluid. Nanofluidics.

[2] Pérez-Luna V.H., González-Reynoso O. (2018). Encapsulation of Biological Agents in Hydrogels for Therapeutic Applications. Gels.

[3] Elkhoury K., Koçak P., Kang A., Arab-Tehrany E., Ward J.E., Shin S.R. (2020). Engineering Smart Targeting Nanovesicles and Their Combination with Hydrogels for Controlled Drug Delivery. Pharmaceutics.

[4] Hegab R.A., Pardue S., Shen X., Kevil C., Peppas N.A., Caldorera-Moore M.E. (2020). Effect of Network Mesh Size and Swelling to the Drug Delivery from PH Responsive Hydrogels. J. Appl. Polym. Sci..

[5] Sood N., Bhardwaj A., Mehta S., Mehta A. (2016). Stimuli-Responsive Hydrogels in Drug Delivery and Tissue Engineering. Drug Deliv..

[6] Nele V., Wojciechowski J.P., Armstrong J.P.K., Stevens M.M. (2020). Tailoring Gelation Mechanisms for Advanced Hydrogel Applications. Adv. Funct. Mater..

[7] Ekladious I., Colson Y.L., Grinstaff M.W. (2019). Polymer–Drug Conjugate Therapeutics: Advances, Insights and Prospects. Nat. Rev. Drug Discov..

[8] Hershfield M.S., Buckey R.H., Greenberg M.L., Melton A.L., Richard S., Hatem C., Kurtzberg J., Markert L., Kobayashi R., Kobayashi L. (1987). Treatment of Adenosine Deaminase Deficiency with Polyethylene Glycol-Modified Adenosine Deaminase. N. Engl. J. Med..

[9] Graham M.L. (2003). Pegaspargase: A Review of Clinical Studies. Adv. Drug Deliv. Rev..

[10] Coyle T.E., Reding M.T., Lin J.C., Michaels L.A., Shah A., Powell J. (2014). Phase I Study of BAY 94-9027, a PEGylated B-Domain-Deleted Recombinant Factor VIII with an Extended Half-Life, in Subjects with Hemophilia A. J. Thromb. Haemost..

[11] Konkle B.A., Stasyshyn O., Chowdary P., Bevan D.H., Mant T., Shima M., Engl W., Dyck-Jones J., Fuerlinger M., Patrone L. (2015). Pegylated, Full-Length, Recombinant Factor VIII for Prophylactic and on-Demand Treatment of Severe Hemophilia A. Blood.

[12] Thomas J., Levy H., Amato S., Vockley J., Zori R., Dimmock D., Harding C.O., Bilder D.A., Weng H.H., Olbertz J. (2018). Pegvaliase for the Treatment of Phenylketonuria: Results of a Long-Term Phase 3 Clinical Trial Program (PRISM). Mol. Genet. Metab..

[13] Landewé R., Braun J., Deodhar A., Dougados M., Maksymowych W.P., Mease P.J., Reveille J.D., Rudwaleit M., Van Der Heijde D., Stach C. (2014). Efficacy of Certolizumab Pegol on Signs and Symptoms of Axial Spondyloarthritis Including Ankylosing Spondylitis: 24-Week Results of a Double- Blind Randomised Placebo-Controlled Phase 3 Study. Clin. Epidemiol. Res..

[14] Baraf H.S.B., Becker M.A., Gutierrez-urena S.R., Treadwell E.L., Vazquez-mellado J. (2013). Tophus Burden Reduction with Pegloticase: Results from Phase 3 Randomized Trials and Open-Label Extension in Patients with Chronic Gout Refractory to Conventional Therapy Tophus Burden Reduction with Pegloticase: Results from Phase 3 Randomized Trials A. Arthritis Res. Ter..

[15] Dijkgraaf E.M., Santegoets S.J.A.M., Reyners A.K.L., Goedemans R., Nijman H.W., van Poelgeest M.I.E., van Erkel A.R., Smit V.T.H.B.M., Daemen T.A.H.H., van der Hoeven J.J.M. (2015). A Phase 1/2 Study Combining Gemcitabine, Pegintron and P53 SLP Vaccine in Patients with Platinum-Resistant Ovarian Cancer. Oncotarget.

[16] Chen X., Chen X., Chen W., Ma X., Huang J., Chen R. (2014). Extended Peginterferon Alfa-2a (Pegasys) Therapy in Chinese Patients With HBeAg-Negative Chronic Hepatitis B. J. Med. Virol..

[17] Kosaka Y., Rai Y., Masuda N., Takano T., Saeki T., Nakamura S., Shimazaki R., Ito Y., Tokuda Y., Tamura K. (2015). Phase III Placebo-Controlled, Double-Blind, Randomized Trial of Pegfilgrastim to Reduce the Risk of Febrile Neutropenia in Breast Cancer Patients Receiving Docetaxel/Cyclophosphamide Chemotherapy. Support. Care Cancer.

[18] Freda P., Gordon M., Kelepouris N., Jonsson P., Koltowska-Haggstrom M., Van Der Lely A. (2015). Long-Term Treatment with Pegvisomant as Monotherapy in Patients with Acromegaly: Experience from Acrostudy. Endocr. Pract..

[19] Autrata R., Krejčířová I., Šenková K., Holoušová M., Doležel Z., Borek I. (2012). Intravitreal Pegaptanib Combined with Diode Laser Therapy for Stage 3+ Retinopathy of Prematurity in Zone I and Posterior Zone II. Eur. J. Ophthalmol..

[20] Macdougall I.C., Robson R., Opatrna S., Liogier X., Pannier A., Jordan P., Dougherty F.C., Reigner B. (2006). Pharmacokinetics and Pharmacodynamics of Intravenous and Subcutaneous Continuous Erythropoietin Receptor Activator (C.E.R.A.) in Patients with Chronic Kidney Disease. Clin. J. Am. Soc. Nephrol..

[21] Kieseier B.C., Arnold D.L., Balcer L.J., Boyko A.A., Pelletier J., Zhu Y., Seddighzadeh A., Hung S., Deykin A., Sheikh S.I. (2015). Peginterferon Beta-1a in Multiple Sclerosis: 2-Year Results from ADVANCE. Mult. Scler. J..

[22] Garnock-jones K.P. (2015). Naloxegol: A Review of Its Use in Patients with Opioid-Induced Constipation. Adis Drug Eval..

[23] Xu Q., Hashimoto M., Dang T.T., Hoare T., Kohane D.S., Whitesides G.M., Langer R., Anderson D.G., David H. (2009). Preparation of Monodisperse Biodegradable Polymer Microparticles Using a Microfluidic Flow-Focusing Device for Controlled Drug Delivery. Small.

[24] Mao S., Guo C., Shi Y., Li L.C. (2012). Recent Advances in Polymeric Microspheres for Parenteral Drug Deliverypart 2. Expert Opin. Drug Deliv..

[25] Danaei M., Dehghankhold M., Ataei S., Hasanzadeh Davarani F., Javanmard R., Dokhani A., Khorasani S., Mozafari M. (2018). Impact of Particle Size and Polydispersity Index on the Clinical Applications of Lipidic Nanocarrier Systems. Pharmaceutics.

[26] Kim J., Vanapalli S.A. (2013). Microfluidic Production of Spherical and Nonspherical Fat Particles by Thermal Quenching of Crystallizable Oils. Langmuir.

[27] Aghaei H., Solaimany Nazar A.R. (2019). Continuous Production of the Nanoscale Liposome in a Double Flow-Focusing Microfluidic Device. Ind. Eng. Chem. Res..

[28] Baby T., Liu Y., Middelberg A.P.J., Zhao C.X. (2017). Fundamental Studies on Throughput Capacities of Hydrodynamic Flow-Focusing Microfluidics for Producing Monodisperse Polymer Nanoparticles. Chem. Eng. Sci..

[29] Neužil P., Giselbrecht S., Huang T.J., Manz A. (2012). Revisiting Lab - on - a - Chip Technology for Drug Discovery. Nat. Rev. Drug Discov..

[30] Jeong H.H., Issadore D., Lee D. (2016). Recent Developments in Scale-up of Microfluidic Emulsion Generation via Parallelization. Korean J. Chem. Eng..

[31] Liu Z., Fontana F., Python A., Hirvonen J.T., Santos H.A. (2020). Microfluidics for Production of Particles: Mechanism, Methodology, and Applications. Small.

[32] Baroud C.N., Gallaire F., Dangla R. (2010). Dynamics of Microfluidic Droplets. Lab Chip.

[33] Baah D., Floyd-Smith T. (2014). Microfluidics for Particle Synthesis from Photocrosslinkable Materials. Microfluid. Nanofluidics.

[34] Xu J.H., Luo G.S., Li S.W., Chen G.G. (2006). Shear Force Induced Monodisperse Droplet Formation in a Microfluidic Device by Controlling Wetting Properties. Lab Chip.

[35] Tan Y.C., Cristini V., Lee A.P. (2006). Monodispersed Microfluidic Droplet Generation by Shear Focusing Microfluidic Device. Sens. Actuators B Chem..

[36] Zhu P., Wang L. (2016). Passive and Active Droplet Generation with Microfluidics: A Review Passive and Active Droplet Generation with Microfluidics: A Review. Lab Chip.

[37] Damiati S.A., Rossi D., Joensson H.N., Damiati S. (2020). Artificial Intelligence Application for Rapid Fabrication of Size-Tunable PLGA Microparticles in Microfluidics. Sci. Rep..

[38] Dang T.D., Kim Y.H., Kim H.G., Kim G.M. (2012). Preparation of Monodisperse PEG Hydrogel Microparticles Using a Microfluidic Flow-Focusing Device. J. Ind. Eng. Chem..

[39] Jo Y.K., Lee D. (2020). Biopolymer Microparticles Prepared by Microfluidics for Biomedical Applications. Small.

[40] Hwang D.K., Dendukuri D., Doyle P.S. (2008). Microfluidic-Based Synthesis of Non-Spherical Magnetic Hydrogel Microparticles. Lab Chip.

[41] Lewis C.L., Lin Y., Yang C., Manocchi A.K., Yuet K.P., Doyle P.S., Yi H. (2010). Microfluidic Fabrication of Hydrogel Microparticles Containing Functionalized Viral Nanotemplates. Langmuir.

[42] Choi C.H., Jung J.H., Hwang T.S., Leezz C.S. (2009). In Situ Microfluidic Synthesis of Monodisperse PEG Microspheres. Macromol. Res..

[43] Yu B., Cong H., Liu X., Ren Y., Wang J., Zhang L., Tang J., Ma Y., Akasaka T. (2013). Preparation of Monodisperse PEG Hydrogel Composite Microspheres via Microfluidic Chip with Rounded Channels. J. Micromechanics Microengineering.

[44] Chen M., Aluunmani R., Bolognesi G., Vladisavljević G.T. (2022). Facile Microfluidic Fabrication of Biocompatible Hydrogel Microspheres in a Novel Microfluidic Device. Molecules.

[45] Kizilel S., Pérez-Luna V.H., Teymour F. (2004). Photopolymerization of Poly(Ethylene Glycol) Diacrylate on Eosin-Functionalized Surfaces. Langmuir.

[46] Acosta-Cuevas J.M., González-García J., García-Ramírez M., Pérez-Luna V.H. (2021). Generation of Photopolymerized Microparticles Based on PEGDA Using Microfluidic Devices. Part 1. Initial Gelation Time and Mechanical Properties of the Material. Micromachines.

[47] Lee C. (2013). Mathematical Modeling of Poly(Ethylene Glycol) Diacrylate Hydrogel Synthesis via Visible Light Free-Radical Photopolymerization for Tissue Engineering Applications. Ph.D. Thesis.

[48] Iannotti V., Ausanio G., Ferretti A.M., Babar Z.U.D., Guarino V., Ambrosio L., Lanotte L. (2023). Magnetic Response of Nano/Microparticles into Elastomeric Electrospun Fibers. J. Funct. Biomater..

[49] Wehking J.D., Gabany M., Chew L., Kumar R. (2014). Effects of Viscosity, Interfacial Tension, and Flow Geometry on Droplet Formation in a Microfluidic T-Junction. Microfluid. Nanofluidics.

